# Supplementary material for: Multi-metal electrohydrodynamic redox 3D printing at the submicron scale
Source: Nat Commun. 2019 Apr 23;10:1853. doi: 10.1038/s41467-019-09827-1 (PMC6479051; doi:10.1038/s41467-019-09827-1)
Supplement: Supplementary file 1 — Supplementary Information [file 41467_2019_9827_MOESM1_ESM.pdf]

Supplementary Materials

Multi-metal electrohydrodynamic redox 3D printing at the submicron scale

Reiser et al.

Supplementary Note 1: Electrical conductivity of substrates

Electrons are required for the reduction reaction involved in the deposition of metals by EHD-RP. Currently, these electrons need to be supplied via charge transfer from the substrate, although alternative sources could be imagined. Consequently, direct printing of metals is easiest on electrically conductive and semiconductive substrates (Supplementary Fig. 1a). Direct printing on electrically insulating materials is not accessible, but printing across insulators between conductive pads is enabled by the as-deposited conductivity of the printed metals (Supplementary Fig. 1b–c).

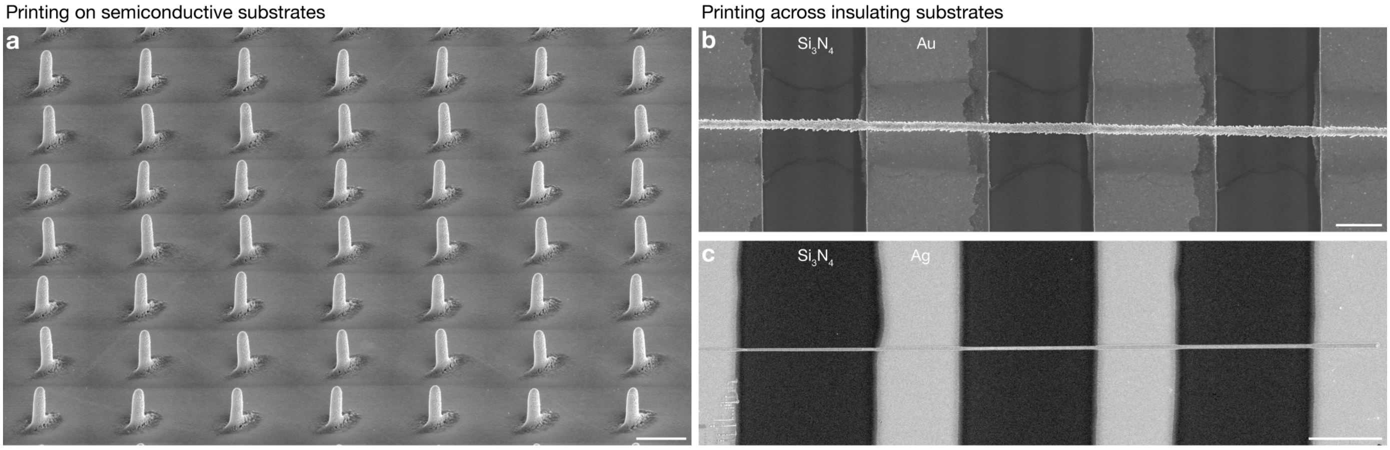

Supplementary Figure 1: **Printing on semiconductive and insulating substrates. a,** Side-view SE SEM micrograph of Cu pillars printed directly onto a single-crystalline Ge substrate (undoped and as-received). Scale bar: 1 μm. **b – c,** Top-view SEM micrographs of Cu lines printed between Ag electrodes across electrically insulating Si_3N_4 substrates: printing from conductive regions onto and across insulating substrates is facilitated by the as-deposited conductivity of the printed lines (SEM signal: a, b: SE, c: BSE). Scale bar b: 2 μm, c: 50 μm.

Supplementary Note 2: Printing Au

Apart from Cu and Ag, we also report printing of Au structures (Supplementary Fig. 2). In comparison with Cu and Ag, the surface morphology of Au pillars suggests a higher porosity with more pronounced amounts of carbon residue.

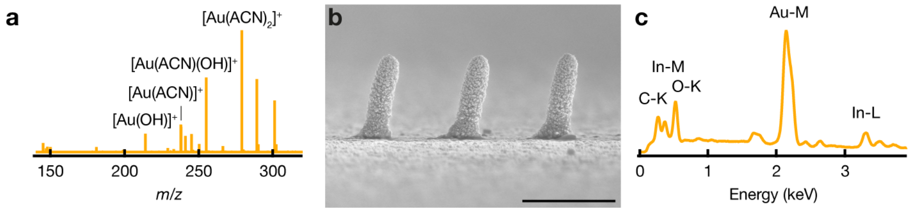

Supplementary Figure 2: **Printing Au.** **a**, Mass spectrum for electrospraying with a Au wire. **b**, SE micrograph and **c**, corresponding EDX spectrum of Au-pillars printed on an ITO substrate. A portion of the C-K signal is presumably related to carbonaceous residues within the pillars. O-K, In-M and In-L signals originate from the ITO substrate. Scale bar: 1 µm. Image tilt b: 82°.

Supplementary Note 3: Effect of nozzle size on printing modes

The use of different nozzle diameters enables three main printing modes: a so termed spraying mode, a focused mode and a porous mode (Supplementary Fig. 3). While we typically use nozzles that facilitate the focused mode, the other modes can be of interest if either thin but homogeneous films need to be deposited (spraying mode) or high porosity is required, e.g., for high surface area or high optical absorption. The focused mode is most suited for high-resolution printing. Here, a nozzle diameter in the range of 160 – 200 nm facilitates focused ejection of ion-loaded solvent. Additionally, solvent flow rates are low enough to allow for prompt evaporation of solvent, resulting in possibly no or only a small sessile solvent droplet on the substrate. Printed features show homogeneous diameters and dense microstructures, and growth is fast and regular. Highly porous structures are achieved with nozzle diameters much larger than 200 nm. In this mode, a clearly visible, sessile solvent droplet is sustained on the substrate by the increased solvent flow rate. As a downside, the lateral reproducibility is reduced and growth rate is irregular. A defocused, spray-like ejection

from nozzles $\leq$100 nm characterises the third mode. As a consequence, lateral feature size is increased and pillars show a large base that gradually narrows due to charge-focusing[1] as pillar growth proceeds.

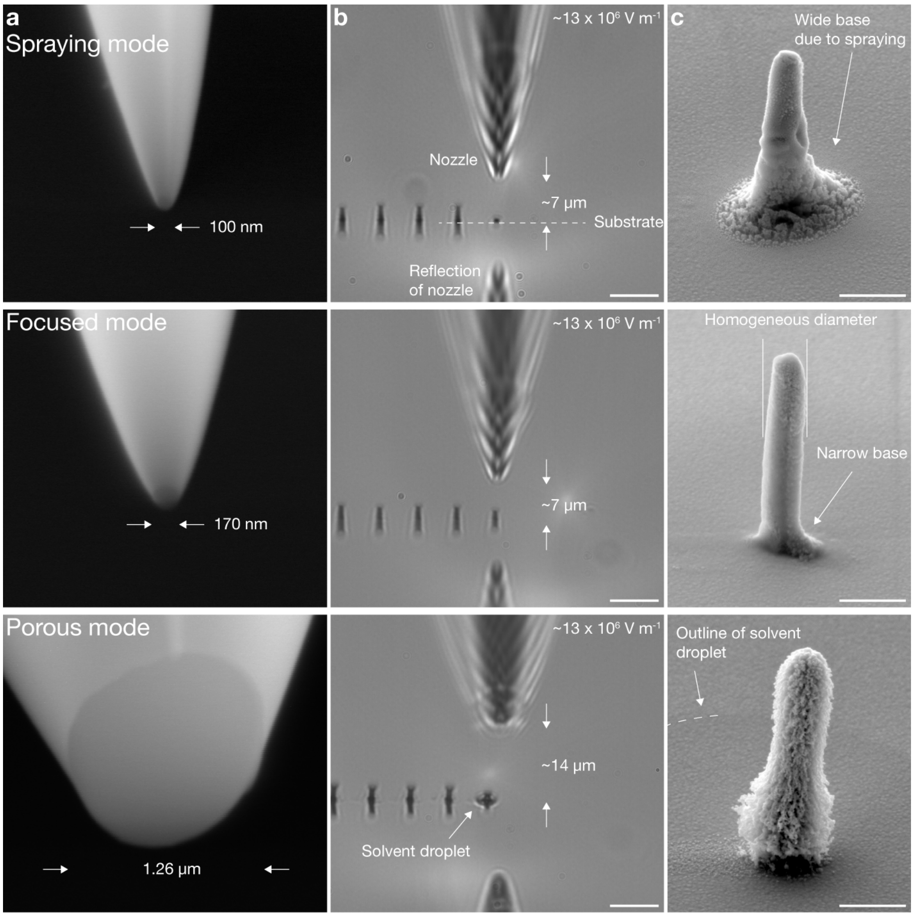

Supplementary Figure 3: **Three printing modes facilitated by different nozzle diameters.** **a**, Low-vacuum SE micrographs of nozzles with three characteristic diameters, **b**, optical images of the printing process at a constant electric field, and **c**, SE micrographs of representative pillars printed with the respective nozzles. Scale bars b: 5 µm, c: 500 nm. **Spraying mode:** spraying of the solvent is typically observed for nozzle openings $\leq$100 nm, resulting in pillars with a pronounced base and inhomogeneous diameters. This mode is best applied for spraying of thin films. **Focused mode:** using nozzles of 160 – 250 nm in diameter, the deposition is focused and pillars grow with an almost homogeneous cross section. This mode is used for high-resolution printing. **Porous mode:** For large nozzles with openings on the order of 1 µm, a higher mass flow feeds a sessile solvent droplet during printing. This results in highly porous deposits and poorer lateral resolution.

Supplementary Note 4: Chemical switching

Reproducibility and alignment

Printing two metals from a single, multichannel nozzle mitigates alignment issues considerably. Consequently, printing structures that demand well aligned deposition of two materials, e.g., the layered pillars shown in Fig. 2, can be printed with high reproducibility (Supplementary Fig. 4). Nevertheless, the asymmetry of a two-channel nozzle can cause some spacial shift of the two materials: in the shown experiment, the alignment upon the Ag-to-Cu switching was ideal, while the switching from Cu to Ag resulted in a slight lateral misalignment of approximately half a pillar-diameter. A similar shift is observed in the printed sequence shown in Fig. 2b. If such a shift occurs, it is typically constant for a given nozzle, and could possibly be corrected by translating the stage accordingly. Yet, the magnitude of the lateral shift between two materials varies from nozzle to nozzle, as the asymmetry of the nozzles is not constant due to uncontrollable variations in the fabrication process. The shift for a typical nozzle, as measured in Fig. 2b, is on the order of 150 ± 10 nm, with a typical line width of 330 ± 20 nm for each of the individual materials. In general, the misalignment is most critical for 2D structures, as the shift is to some extent mitigated by the auto-focussing effect of EHD-deposition[1]. Nevertheless, a more reproducible nozzle-fabrication routine or a re-design of the nozzle should be considered to enable more reproducible alignment of the two materials.

Inhomogeneities in the wall in Fig. 2d and in the line sequence in Fig. 2b are caused by small deviations in growth speeds of the two metals: in both cases, Ag was printing at a slightly slower rate. Thus, the parts of the wall in Fig. 2d that contain Ag layers (middle) are approx. 30% lower in height compared to the sections of the wall that are built from Cu only (sides). To improve the geometrical reproduction of the designed features, a feedback-loop for the individual growth speeds based on the electric current through the separate sources should be implemented in the future.

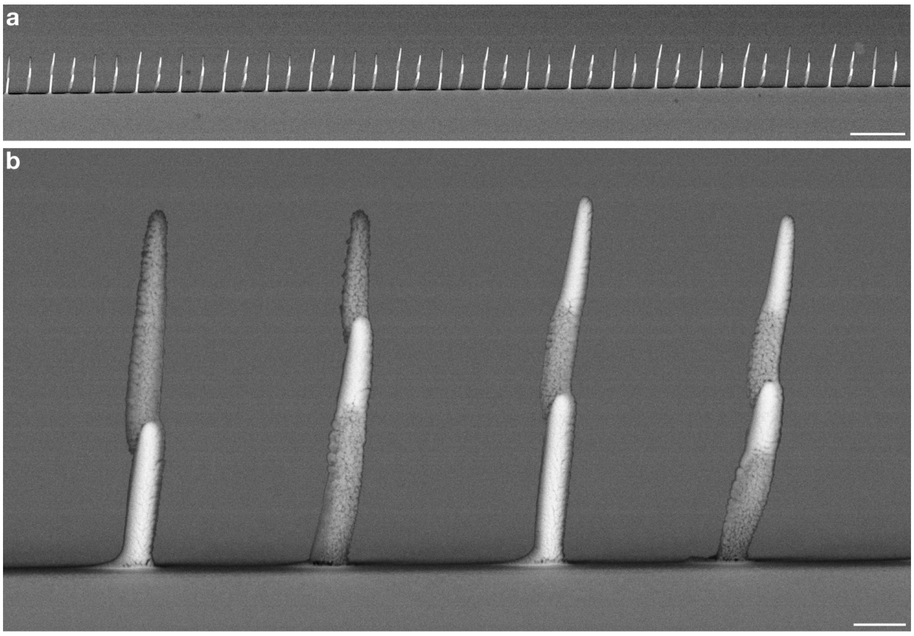

Supplementary Figure 4: **Reproducibility of the chemical switching.** **a**, BSE micrograph of a sequence of chemically layered pillars. Bright contrast corresponds to Ag, dark contrast to Cu. Scale bar: 5 μm. **b**, Zoomed image that shows the repetitive unit of the sequence in (a). This micrograph corresponds to the SE image and the EDX elemental maps shown in Fig. 2c of the main text. Scale bar: 500 nm.

Switching speed

Clear and complete switching between two ejected species of ions is the prerequisite for a small chemical feature size when modulating the chemistry on the fly. A square-wave pulse train is thus the desirable ion current profile upon switching: fast rise and fast drop of the current guarantee a spatially most confined chemical voxel. The typical electrical setup for printing consists of a polarised electrode connected to the high output of a voltage source, and a grounded substrate (Supplementary Fig. 5a). To stop the ion ejection from the nozzle, the electrode needs to be depolarised. This can be achieved by either setting the electrode to an electrically floating or grounded state. If the electrode is switched into a floating state, the surface charge is removed through further oxidation of the electrode and ejection of ions through the nozzle until the surface potential is equilibrated. Thus, the ejection ceases in a slow and gradual manner (Supplementary Fig. 5b). In contrast, if the electrode is grounded, all surface charge is removed through the external circuitry, and the

ejected ion current is quenched rapidly.

If multiple electrodes are used as ion sources, any electrode switched off must be in an electrically floating state to avoid unwanted redox-currents in-between the individual electrodes (the individual channels of the nozzle are connected by the solvent across the meniscus at the orifice). Consequently, the easiest strategy to switch between two electrodes is to alternate the individual sources from high to floating. As described above, this approach results in prolonged ejection of ions after turning off an electrode. Consequently, switching between two elements is blurred (Supplementary Fig. 5c). To obtain a square-wave ion-current output, the electrode to be turned OFF needs to be grounded just long enough to enable complete removal of the surface charge through the external circuit (Supplementary Fig. 5d). Subsequently, this electrode can be switched to a floating OFF state before the other electrode is polarised. This approach necessitates the intermittent off-

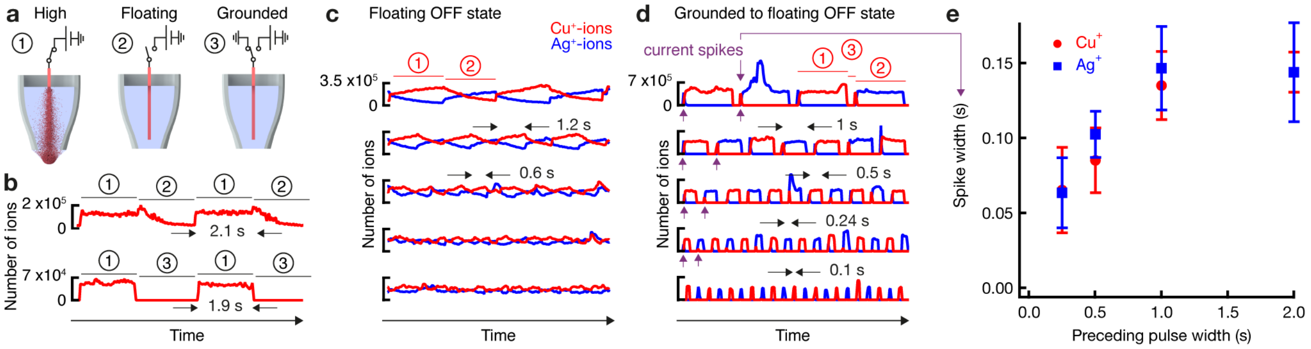

Supplementary Figure 5: **Increased switching speed by removal of surface charge.** **a**, To stop the ejection of ions from a nozzle, the high potential of the electrode (1) needs to be removed. This may either occur by switching the source into an electrically floating (2) or a grounded (3) OFF state. In the floating case, the electrode's remaining charge is removed through extended ion ejection. In the grounded case, the charge is removed through the external circuit. **b**, MS ion currents recorded for a Cu electrode switched from high (1) to floating (2) (top) or from high (1) to grounded (3) (bottom). The numbers indicate the switching-states. **c – d**, MS ion currents recorded for switching between a Cu (red) and a Ag (blue) electrode at decreasing intervals, either by switching the sources directly into the floating OFF state (**c**) or only after an intermittent grounded stage (**d**). The numbers indicate the switching-states for the red current transient. Current spikes of the opposite ion species are observed upon switching in (d) (purple arrows). **e,** Width of the current spikes (measured at their base) as a function of the preceding ON-time of the respective ion source (Cu^+: red circles, Ag^+: blue squares). The error bars indicate the standard deviation of the measurement.

time of 100 ms between deposition pulses. The interrupted deposition could be corrected for by synchronised pausing of stage translation.

While the switching mode high – grounded – floating enables faster switching, it does not avoid a current spike of the opposite ion species at the onset of each ion pulse (purple arrows in Supplementary Fig. 5d). These spikes presumably indicate the purging of ions from the OFF channel of the printing nozzle – ions that were dissolved but not yet ejected during the preceding pulse. This ejection of previously ionized ions limits the sharpness of any interface produced by switching between two metals and will result in a gradient region containing a mixture of both metals. The width of this interface can be estimated by the approximate pulse duration of the current spikes: Supplementary Fig. 5e plots the width of the current spikes, measured at their base, as a function of the preceding ON-time of the respective source. The observed maximum spike width of 0.15 s translates into a width of 100 – 200 nm for a resulting gradient region when assuming a typical printing speed of 1 $\mu m\ s^{-1}$. As this is on the order of the lateral printing resolution, the purging of the leftover ions does currently not limit the minimal in-plane interface width (as it is also limited by the lateral resolution). In contrast, the spike width limits the width of interfaces when printing out of plane.

Supplementary Note 5: Geometry

A typical feature size of 200 nm is achieved when printing with a single-channel nozzle. Additionally, lines and pillars of smaller diameters were printed (Supplementary Fig. 6a – b). The largest aspect ratio printed to date was >1380 (Supplementary Fig. 6c). The inhomogeneous diameter of the wire was caused by manually retracting the stage. High-resolution SEM images could only be recorded at the base of the wire, since the upper part of the wire oscillates in the electron beam.

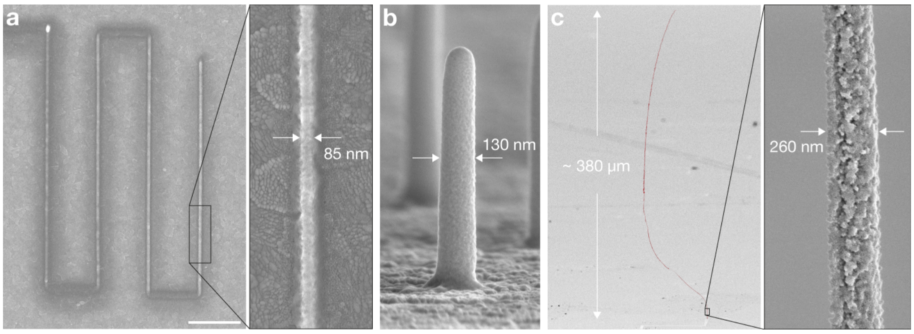

Supplementary Figure 6: **Smallest feature size and highest aspect ratio.** SE micrographs of **a**, a <100 nm wide Cu line printed on ITO. Scale bar: 2 µm. **b**, A Cu pillar 130 nm in diameter. Image tilt: 77°. **c**, A Cu wire of aspect ratio >1,380. Image tilt: 80°.

Geometrical fidelity and reproduction of designed features is critical for future applications. Unwanted growth at edges and corners (Fig. 2d and 3b) and elevated portions of a geometry is favored by the auto-focussing effect of EHD-printing. Schneider et al.[2] have shown that this effect could be avoided by adjusting the print strategy – an approach that we have not implemented yet. Further, the local growth rate is a function of the nozzle-substrate distance. Thus, precise control of the distance to previously printed features and non-planar substrates will be crucial for improved geometrical control. A feedback loop that adjusts the gap-distance in-situ based on the reduction current, representing the local growth rate, could help to improve geometrical reproduction.

Supplementary Note 6: Printing Speed

Typical out-of-plane printing speeds of EHD-RP are on the order of 3 – 5 voxels per second (normalised by the X-Y feature size) for minimal feature sizes in the range of 100 – 250 nm. Higher growth rates are physically possible when increasing the printing voltage. Maximum out-of-plane printing speeds that still allowed for a reliable measurement were 10 voxels s^{-1} (Supplementary Fig. 7a). Yet, the higher electric field increases the feature size to 300 – 500 nm, and the printed pillars show a coarser topology due to the influence of the electric field on the microstructure (Supplementary Fig. 11). In plane, printed lines become discontinuous due to insufficient coverage of the deposited material at printing speeds $>5 \mu m\ s^{-1}$ when using standard ejection voltages. At the other end of the spectrum, lowest growth rates explored were in the range of 0.2 – 0.5 voxel s^{-1} (out-of-plane). Such decreased rates are of interest if a small out-of-plane chemical voxel size is required (min. chemical voxel size = max. switching frequency $\times$ min. growth rate).

A speed of 3 – 10 voxels s^{-1} outperforms other electrochemical techniques by one order of magnitude (in average, Supplementary Fig. 7b), while competing with their very best feature-sizes. The increased speed facilitates the additive, electrochemical fabrication of structures with at least two dimensions in the sub-millimeter range: a diffraction grating of 300×300 individual Cu pillars printed with a residence time of approximately 200 ms per pillar is printed in a total time of approximately 6 h (Supplementary Fig. 7c – d, Supplementary Movie 2).

Compared to other, non-electrochemical small-scale AM methods, the speed of EHD-RP is average (Supplementary Fig. 7b). While parallelisation with multi-nozzle printheads could enhance the throughput as demonstrated for EHD-printing[5,6], the moderate speed raises the question after the limiting factors that cap the maximum attainable speed of EHD-RP. Ultimately, the method might be limited by one of the three fundamental processes that define the ion current: ion generation, ion transport and ion reduction. In prac-

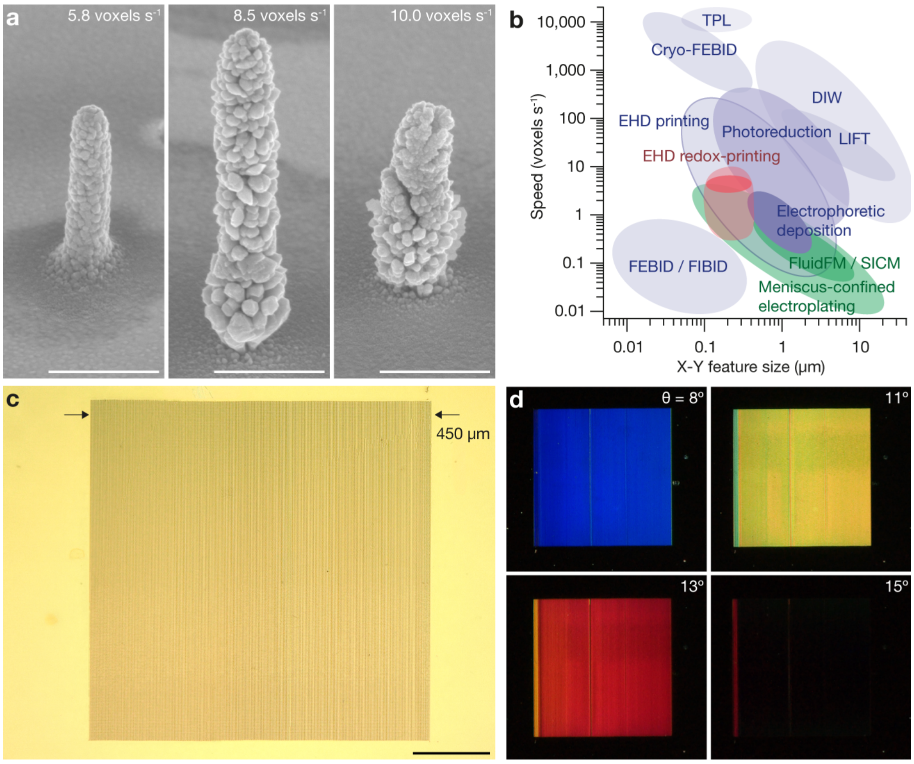

Supplementary Figure 7: **Speed facilitates printing of sub-millimeter-scale objects.** **a**, Cu pillars printed at average out-of-plane growth speeds of 5.8 to 10.0 voxel s^{-1}. Respective residence times: 625, 639, and 257 ms. Scale bars: 500 nm. Image tilt: 57°. **b**, Speed versus X-Y feature size of contemporary small-scale metal AM techniques, with EHD-RP represented in red (dark red: typical speeds used in this report). A maximum speed on the order of 10 voxel per second is one order of magnitude faster than previous electrochemical techniques (green). Adapted with permission[3]. Copyright 2017, John Wiley and Sons. Additional datapoint for two-photon lithography (TPL) from reference[4]. **c**, Optical bright field micrograph of a diffraction grating built from 300×300 Cu pillars printed with a residence time of approximately 0.2 s per pillar. Total printing time was approximately 6 hours. Scale bar: 100 μm. **d**, Optical dark field micrographs showing the first order of diffraction of the grating in (c). The angles indicate the grating angle θ relative to the position of the grating when light is reflected in zero order.

tice however, practical considerations limit the maximum speed, as increased ejection voltages necessary for higher speed show some negative influence on the printed structures: first, a higher ejection voltage seems to increase the width of printed features (Supplementary Fig. 7a). Second, the enhanced grain growth observed at higher fields (Supplementary Fig. 11) increases the roughness of the printed metals and results in pronounced voids between individual crystallites (Supplementary Fig. 7a). Further, the accuracy of the

deposition as well as the reproducibility of the growth speed are diminished at higher ejection voltages. We speculate that all these effects are related to the formation of a sessile solvent droplet on the substrate that forms at high ejection voltages due to the increased solvent flow rate (Supplementary Fig. 8). Its presence is the current practical limit to higher growth speeds, although its influence on the growth mode and the resulting morphology and voxel size cannot be pinpointed at the moment. Consequently, increasing the evaporation rate of the solvent to avoid the sessile droplet should be considered when aiming for increased speeds, for example by mild heating of the substrate.

We cannot predict a single fundamental factor that will ultimately limit the speed of EHD-RP at this stage. Potential candidates for limiting the absolute printing speed are: the ion reduction rate on the substrate (including charge transfer rate and ion transport rate through the sessile solvent), the ion emission rate available from EHD-ejection modes[7,8] that are suitable for high-resolution printing (i.e., focussed modes), the ion transport rate and the maximum solvent flow rate within the nozzle, and the maximum evaporation rate of solvents suitable for printing. While we already have measured an increase of the emission current by approx. an order of magnitude for jetting modes compared to the standard printing mode (Supplementary Fig. 8c), any other limiting factors have not yet been explored. A factor that is probably not rate limiting is the ion generation at the anode, as the surface area of the anode exceeds the area of the cathode by many orders of magnitude (approx. by a factor of 10^8 if the immersed anode surface is considered, and approx. 10^5 – 10^6 if only the anode area closes to the nozzle apex is considered).

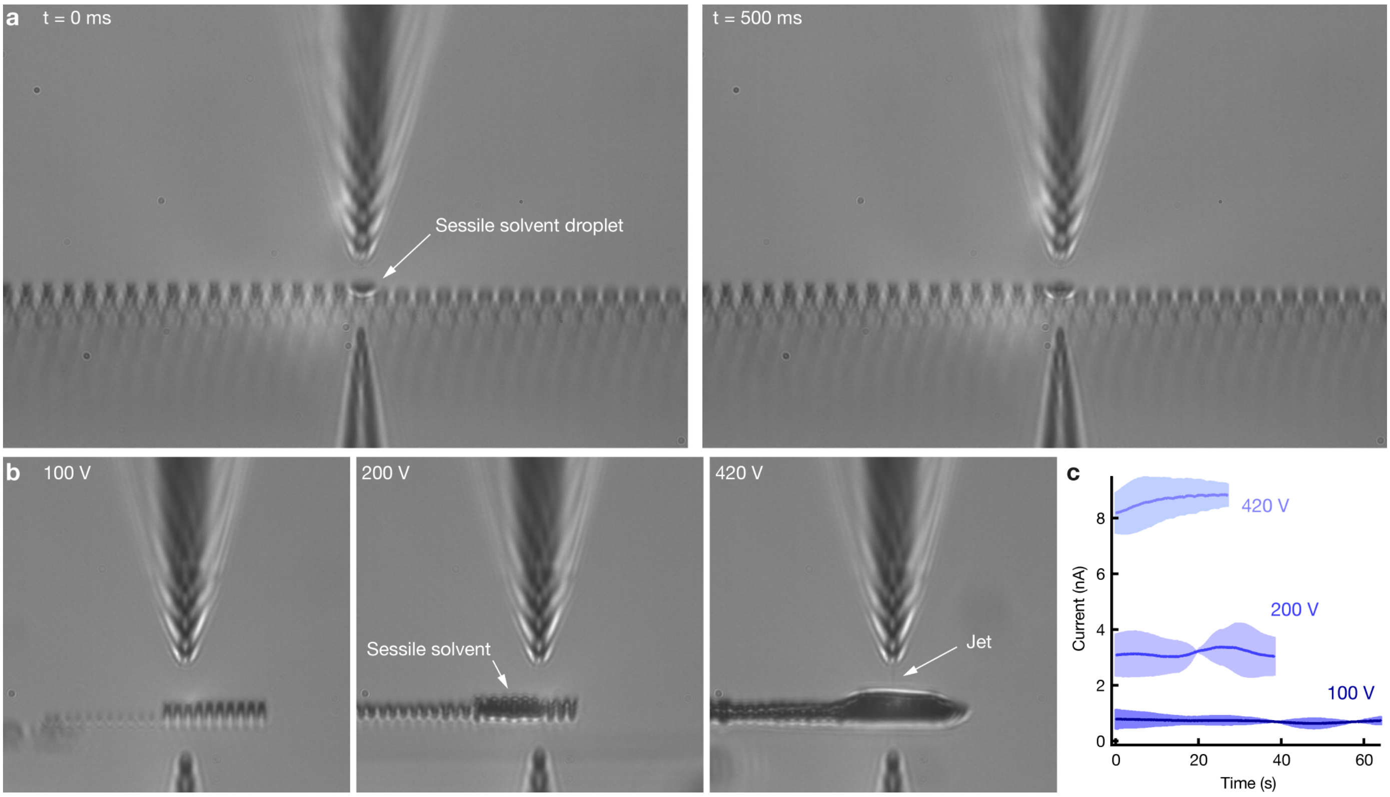

Supplementary Figure 8: **The sessile solvent droplet at high ejection voltages. a,** Sequential optical images of the printing of pillars at 10 voxels s^{-1} (same pillars as in Supplementary Fig. 7a). At high ejection voltages, a sessile solvent droplet is maintained on the substrate. **b,** optical images of the printing of arrays of pillars at increasing ejection voltages. The tip-substrate gap is approx. 8 µm. The formation of a jet, accompanied by a high solvent flow rate, is observed at the highest ejection voltage. **c,** Current transients recorded during the printing of the arrays in b (raw data (light blue) and smoothed data (dark blue)).

Supplementary Note 7: Microstructure

The microstructure of as-deposited metals is polycrystalline and of high density. Printed Cu pillars can be completely dense (Fig. 3g), and an estimation of the pore area fraction of Cu, Ag and Cu-Ag pillars yields densities of >90% for all materials (Supplementary Fig. 9). We assume that the as-deposited, co-printed Cu-Ag material is in a metastable, solid solution state, since it is deposited at room temperature using relatively high growth rates. No evidence for a phase separation of the thermodynamically immiscible Cu-Ag system was detected at length scales accessible with BSE microscopy.

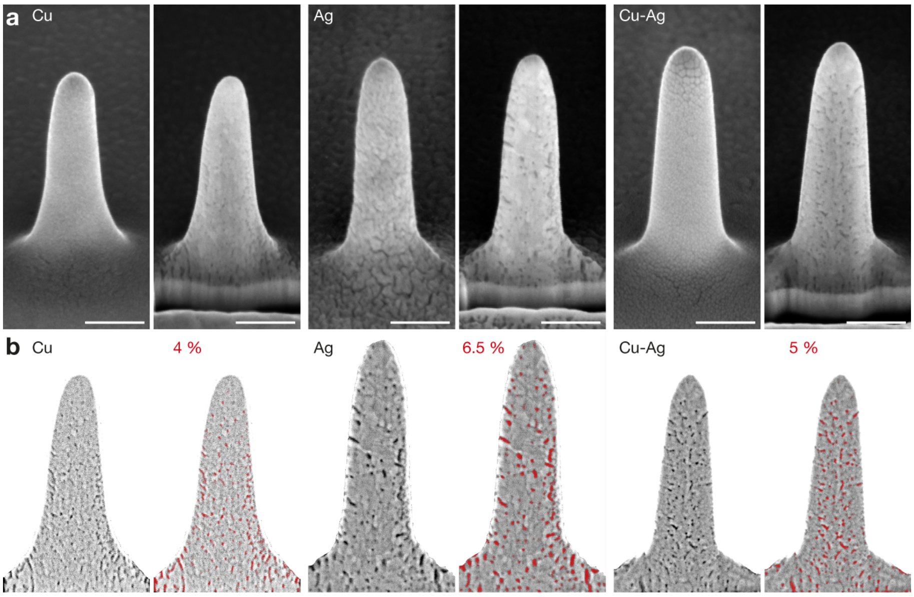

Supplementary Figure 9: **Internal microstructure and pore fraction. a**, InLens SEM micrographs and FIB cross sections of as-deposited Cu, Ag and Cu-Ag pillars printed from the same two-channel nozzle (in each case the rightmost pillar of Fig. 1e in the main text). All pillars are polycrystalline. Scale bars: 200 nm. **b**, The pore area fractions estimated by image contrast analysis are: Cu: 4%, Ag: 6.5%, Cu-Ag: 5%. All images are tilt-corrected.

In general, printing in Ar-atmosphere (100 – 200 ppm O_2) hinders the formation of unwanted oxide phases that could form when printing in air (Supplementary Fig. 10). However, the microstructure analyses by EDX and HAADF imaging both indicate the presence of impurities, presumably due to residual solvent. The pres-

ence of carbon residues is more pronounced in Ag and Au (Fig. 1e and Supplementary Fig. 2c). In the case of Cu, the amount of C is typically below the detection limit of EDS SEM (Fig. 1e).

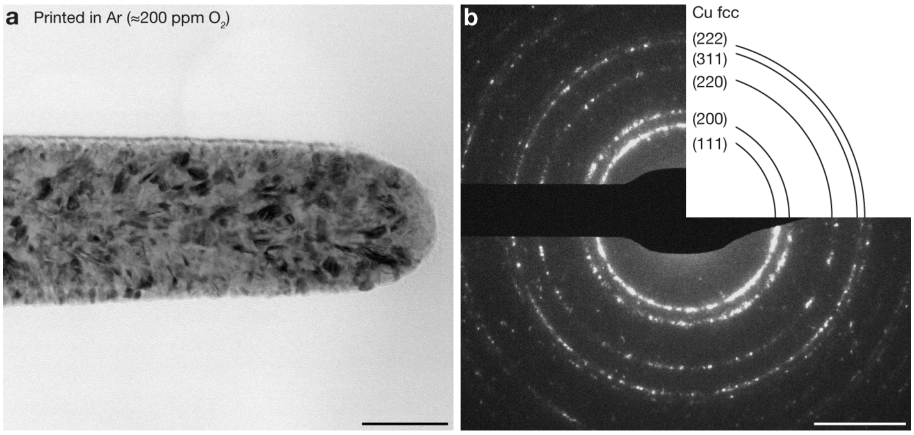

Supplementary Figure 10: **Printing without crystalline oxides.** Cu can be deposited without crystalline oxides if printing is performed in oxygen-deficient atmosphere. **a**, BF-TEM and **b**, corresponding diffraction pattern of a Cu pillar printed Ar atmosphere (≈200 ppm O_2). The electron diffraction pattern shows the Cu fcc structure. Scale bar: a, 100 nm, b: 5 nm^{-1}.

The grain size and defect density is a function of the electric field applied during printing (Supplementary Fig. 11). At low fields of 1×10^7 Vm^{-1}, the grain size is of the order of 20 nm, i.e., approximately a tenth of a pillar diameter. Additionally, the presence of point defects is pronounced (vacancy clusters or nanometer-sized pores, visible as small, dark areas arranged as chains in the atomic number sensitive HAADF STEM micrograph, arrows in Supplementary Fig. 11a). Printing at higher fields, i.e., 4×10^7 V m^{-1}, results in much larger grains, with some grains extending across the whole pillar diameter (≈200 nm in Supplementary Fig. 11b – c). In addition, a crystallographic correspondence between neighbouring grains, i.e. texture, as well as a decrease in point defect density were detected in high-field pillars. The increase in grain size with the electric field as well as the marked decrease in the defect density suggest a pronounced increase of the atomic mobility with the electric field applied during printing.

Gradient microstructures were observed in pillars that were grown under conditions that offer a large variation of the electric field strength during growth, i.e., when a pillar was grown across significantly more than half the gap distance. Under normal printing conditions, the microstructure along the pillar axis is constant (Fig. 3g, Supplementary Fig. 11).

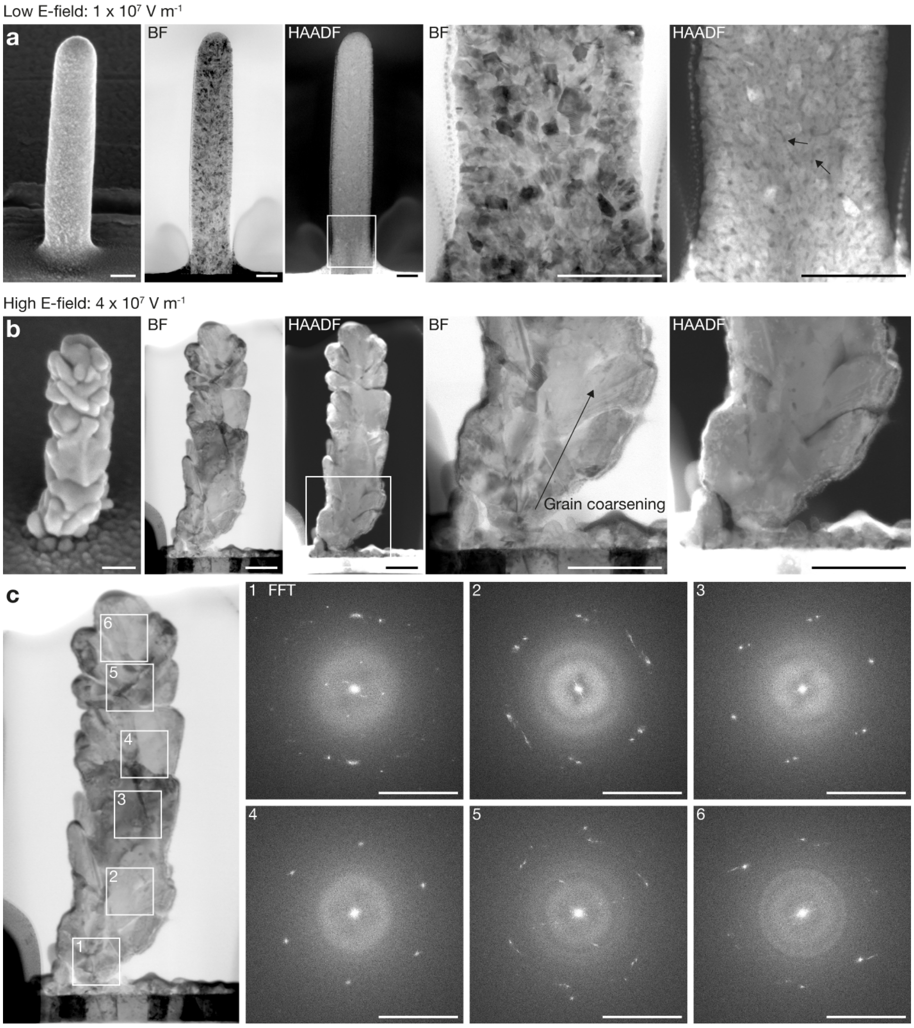

Supplementary Figure 11: **Microstructure as a function of the electric field.** SEM and STEM micrographs of a Cu pillar printed in **a**, low and **b**, high electric field conditions. Scale bars: 100 nm. A comparison of the bright field (BF) micrographs acquired from low- and high-field pillars shows that the crystallite size increases with the electric field: pronounced grain coarsening of the crystallites is observed in (b), while the crystallite size is constant in (a). High-angular annular dark field (HAADF) images show dense microstructures. In the low-field pillars, chains of point defects (vacancy clusters or nanoscale pores) are observed (arrows). The HAADF STEM of the high-field pillar show similar defects, but markedly decreased in number. **c**, Fast Fourier transforms (FFT) of high-resolution BF-TEM images taken at the indicated locations 1 – 6 of the high-field pillar (same pillar as in (b)). The crystallographic orientation of individual grains is aligned, i.e., the pillar is textured. Scale bars: 5 nm^{-1}.

Supplementary Note 8: Materials properties

The nanocrystalline microstructure results in excellent mechanical and good electrical properties of as-printed Cu. A flow strength on the order of 1 – 1.5 GPa and a Young's modulus of 81±9 GPa have been determined by micropillar compression (Supplementary Fig. 12). Especially the strength compares well with the highest literature values for nanocrystalline Cu[9]. The Young's modulus is slightly lower than literature values of 117 – 131 GPa[10], which is possibly caused by residual organic impurities.

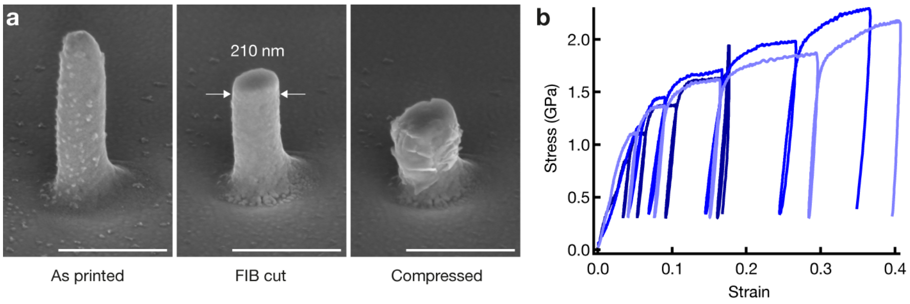

Supplementary Figure 12: **Mechanical properties.** **a**, SE SEM micrographs of an exemplary Cu pillar as deposited (left), FIB cut (middle) and compressed (right). Scale bars: 500 nm. Image tilts: 60°. **b**, Stress-strain curves recorded from three FIB-cut pillars. An unloading Young's modulus of 81 ± 9 GPa and a flow stress in the range of 1 – 1.5 GPa is derived from these curves.

The crystalline and percolated as-deposited microstructure renders as-printed materials electrically conductive. As a result, printing across insulating substrates is enabled if printing starts from a grounded pad (Supplementary Fig. 1b – c, Supplementary Fig. 13) – as-printed geometries guarantee sufficient charge transport for electrochemical reduction. Best as-deposited electrical conductivities were $\approx 0.1\times$ bulk conductivity of Cu. As an example, the electrical resistivity of the Cu line in Supplementary Fig. 13 is $14.4 \pm 2.1 \times 10^{-8}$ Ωm, i.e., $\approx 8.4 \times$ bulk resistivity or $0.12 \times$ bulk conductivity of Cu (1.71×10^{-8} Ωm at 25°C[11]). This value was extracted in a two-point measurement as follows: two Pt-pads for electrical contact were FIB-deposited, once on the grounded Ag electrode (pad 2) and once onto the far end of the line (pad 1). A W-micromanipulator positioned on the pads was used as the high contact, whereas the Ag

electrode was electrically grounded. The resistance of the circuit R_{circuit} (as measured from pad 2) and the resistance of the line plus the circuit $R_{\text{circuit+line}}$ (measured from pad 1) were derived by averaging all data points from three independent current-voltage curves (Supplementary Fig. 13c): $R_{\text{circuit}} = 61.8 \pm 10.7\ \Omega$, $R_{\text{circuit+line}} = 266.8 \pm 10.4\ \Omega$. The resistance of the line was thus calculated as $R_{\text{line}} = R_{\text{circuit+line}} - R_{\text{circuit}} = 205.0 \pm 14.9\ \Omega$. To evaluate a value for the resistivity, we measured the length l of the line in the SEM (Supplementary Fig. 13a), and estimated the average cross-sectional area A of the line from thirteen AFM line profiles recorded at evenly distributed intervals along the length of the line. For $l = 45 \times 10^{-6}$ m and $A = 3.16 \pm 0.40 \times 10^{-14}$ m^2, the above reported resistivity of $\rho_{\text{line}} = (R_{\text{line}}\ A)/l = 14.4 \pm 2.1 \times 10^{-8}\ \Omega$m is calculated.

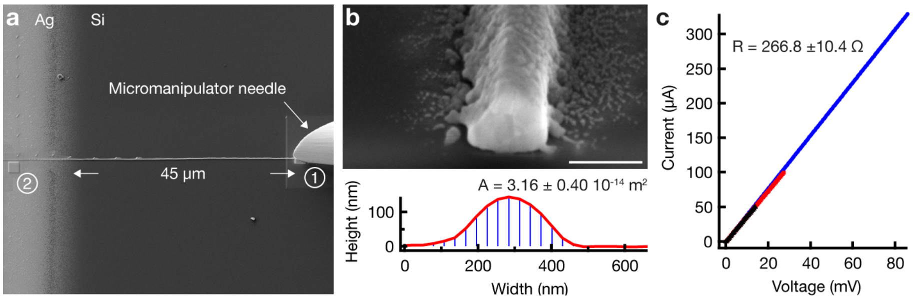

Supplementary Figure 13: **As-deposited conductivity enables printing across electrically insulating substrates.** **a**, SE SEM micrograph of a Cu line printed from a Ag electrode onto a Si wafer covered with a 50 nm thermal oxide and 50 nm Si_3N_4. For resistivity measurements, electrical contact is established using a micromanipulator W needle placed on FIB-deposited Pt pads (1 and 2). **b**, FIB-cut cross section and AFM line profile of the line shown in (a). The cross section was cut just right of pad 1. Channeling contrast reveals a polycrystalline as-deposited microstructure with a grain size <100 nm. Scale bar: 200 nm. Micrograph is tilt-corrected. **c**, Three current-voltage curves measured from pad 1. Subtracting the resistance of the measurement circuit, as recorded from pad 2, derives a resistance of $205.0 \pm 14.9\ \Omega$ for the printed line. This corresponds to a resistivity of $\approx 8.4 \times$ the bulk resistivity of Cu.

A major contribution to the increased resistivity as compared to bulk values is the grain size of <100 nm: compared to a resistivity of a sputtered and annealed Cu film with a grain size comparable to the printed line ($\rho_{\text{PVD}} \approx 4 \times 10^{-8}\ \Omega$m for a grain size of 50 nm and a film thickness of 50 nm[12]), the resistivity of the

as-printed line is only a factor $\rho_{\text{line}}/\rho_{\text{PVD}} \approx 4$ higher. Additional components that contribute to the lowered as-deposited conductivity are the high density of point defects (Supplementary Fig. 11) and probably residual carbon impurities.

In general, it should be noted that the materials properties reported here are obtained from samples printed with not-yet fully optimised printing parameters. Presently, low fields are employed for printing, because they guarantee lower surface roughness and easier control. Nevertheless, the pronounced dependency of the synthesised microstructure on the electric field offers an excellent handle to control and optimise printed microstructures and will allow to adjust global or even local properties to a given application in the future.

Supplementary Discussion 1: Deposition mechanism – electrochemical growth via reduction of ions by charge transfer from the substrate

A key feature of EHD-RP, as claimed in this report, is the electrochemical production of solvated ions and their electrochemical reduction on the substrate. In the following, we show evidence for this process and consequently establish an electrochemical growth mechanism underlying EHD-RP.

Of the two major stages of deposition, the oxidative generation of solvated ions from sacrificial electrodes is widely accepted[13,14], and has previously been verified in electrospray ionisation experiments[15–18]. In contrast, the reduction of ejected ions to elemental metal has received less attention. In principle, two basic mechanisms for the deposition of metallic materials from ejected metal ions can be distinguished: neutralisation of ions on the substrate, followed by the bottom-up growth of a metallic deposit, or neutralisation of ions in flight, followed by cluster formation and deposition of individual particles on the substrate. The first mechanism is supported by Cooks and coworkers: they suggest the neutralisation of solvated ions on the grounded substrate after studying the delocalised synthesis of metals via spraying of metal cations from sacrificial sources[17,18]. Yet, the authors only demonstrate the deposition of isolated nanoparticles and do not present evidence for fused and dense metallic materials. The second mechanism is regularly applied for the synthesis of inorganic nanoparticles by electrospray deposition[19], but usually requires active neutralisation of droplets in flight, followed by pyrolysis of the neutralised precursors (often metal salts). To our knowledge, formation of metallic particles without active neutralisation has not been reported. Nevertheless, both mechanisms are considered for the following discussion of the deposition mechanism underlying EHD-RP. Thus, the main question to be answered is: are ions reduced on the cathode and thus facilitate an electrochemical growth from the substrate, or are the ions neutralised in mid-air and deposit as loose particles onto the substrate?

Our interpretation is primarily based on a comprehensive microstructure analysis of the printed deposits and a detailed look at the nature of the ejected ions. Further support of the growth hypothesis is presented in Supplementary Discussion 2: a theoretical calculation of the maximum size of a particle formed in flight, and a study of the printing on substrates that inhibit neutralisation via charge transfer from the substrate.

The polycrystalline, textured as-deposited microstructure excludes a particle-based deposition mode altogether (Fig. 3g, Supplementary Fig. 9 – 11). Indeed, the microstructure evolution within individual pillars along the pillar axis – grain coarsening and faceted grain boundaries accompanied by the crystallographic alignment of the grains – evidences grain formation and growth during deposition. This is best demonstrated by pillars printed in high field conditions (Supplementary Fig. 11b – c). Pillars fabricated at lower electric fields present less obvious but nevertheless clear evidence of the same growth process occurring at somewhat lower rates. The excellent mechanical strength shown by grown pillars also excludes particle-based materials (Supplementary Fig. 12). Thus, we conclude a mechanism based on electrochemical growth and exclude any significant contribution of particle deposition.

The electrochemical deposition is likely facilitated by the arrival of individual, solvated ad-ions on the substrate. MS data for a large range of m/z evidences the exclusive formation of mono-metallic clusters of ions containing a single metal cation only (Supplementary Fig. 14). The absence of bi- and tri-metal clusters again indicates a very low probability for the formation of any particles: if particles were to nucleate in air, multi-metal clusters, which are intermediate stages of particle formation, must be present in the MS spectra. It should be noted that results from MS are not directly comparable to the printing process, because the time of flight in air is longer in an MS-experiment compared to printing (distance and voltage MS: 0.5 – 2 mm, 150 – 400 V, printing: 5 – 10 μm, 80 – 150 V). Nevertheless, because of the increased flight-time in MS, it can be stated with high certainty that if single-metal ion-clusters are the only species detected in MS, they are also the only species contributing to the growth upon printing.

In summation, we conclude that EHD-RP is based on the electrochemical growth of metals enabled by single-metal ad-ions that are neutralised upon charge transfer from the substrate.

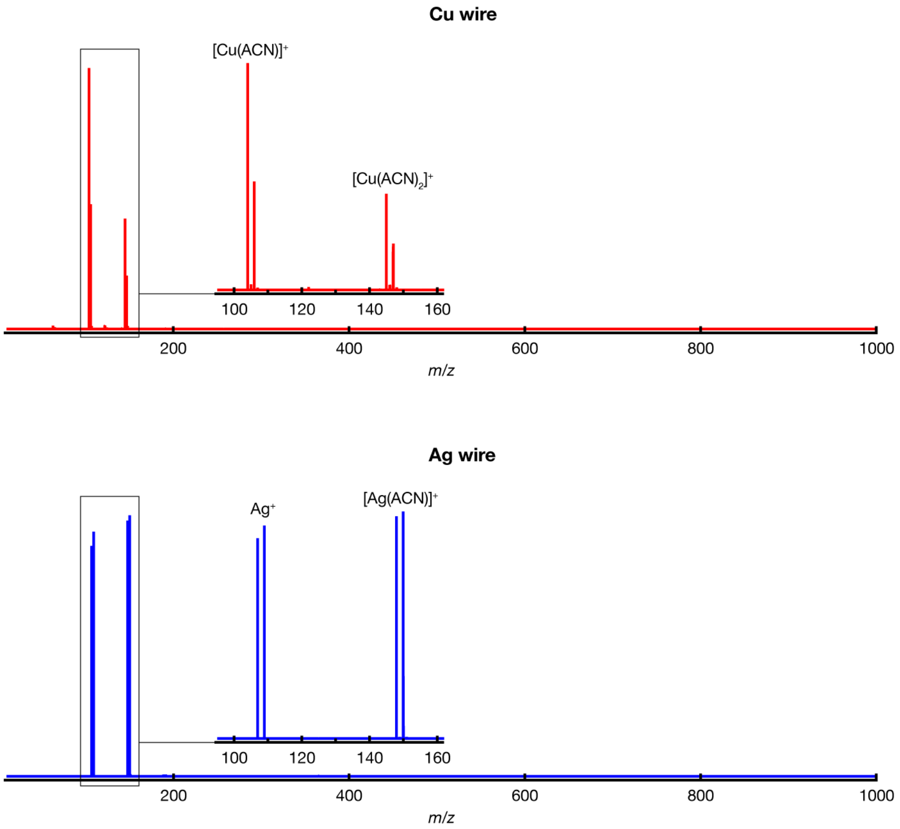

Supplementary Figure 14: **Only single-metal ion clusters are detected in MS.** Mass spectra for spraying with a Cu (top) and a Ag electrode (bottom) immersed in acetonitrile (ACN). No significant contributions other than those from single-metal ion clusters are detected up to a m/z ratio of 1000.

Supplementary Discussion 2: Deposition mechanism – Appendix

Additional arguments that support the hypothesis of an electrochemical growth mechanism are presented in the following: the size of grown crystallites is larger than the theoretical maximum size of particles formed in flight; if charge transfer from the substrate is blocked, no growth occurs.

Maximum theoretical size of crystallites formed in flight

We can estimate a maximum size of particles that could form in flight. If printed structures contain particles or crystallites larger than this critical size, then the features must have grown from the substrate rather than been deposited onto the substrate. To calculate a maximum size, we first assume that a particle will only form from metal cations contained in an individual droplet, i.e., that no ions from multiple droplets combine. This assumption is reasonable since droplets of equal sign of charge will fission and repel each other rather than fuse[20]. The maximum charge that can be contained in a droplet of radius r is given by the Rayleigh limit q_R:

$$q_\mathrm{R} = 8\pi\sqrt{\epsilon_0 \gamma r^3} \tag{1}$$

where ϵ_0 is the permittivity of free space and γ is the surface tension of the liquid. The diameter of the largest droplet that could be ejected from a nozzle is assumed to be the diameter of the nozzle itself. For most cases, this is an unrealistic assumption, since actual droplet diameters are usually a fraction of the nozzle size[1,21,22]. Nevertheless, the hypothesis is acceptable for the argument presented here, because it will lead to an overestimation of the maximum particle size. For acetonitrile, with $\gamma = 0.0291$ N m^{-1}[23], and a typical nozzle diameter of 200 nm, i.e., $r = 100$ nm, q_R is

$$q_\mathrm{R} = 8\pi\sqrt{8.85 \times 10^{-12}\ \mathrm{F\ m^{-1}} \cdot 0.0291\ \mathrm{N\ m^{-1}} \cdot (100 \times 10^{-9}\ \ \mathrm{m})^3} = 4.03 \times 10^{-16}\ \mathrm{C} \tag{2}$$

Faraday's law of electrolysis describes the volume V deposited from a charge q as

$$V = \frac{1}{\rho}\left(\frac{q}{F}\right)\left(\frac{M}{z}\right), \tag{3}$$

with F the Faraday's constant, ρ the density, M the molar mass, and z the valency number of the cation to be deposited. With ρ and M of Cu, $q = q_{\mathrm{R}}$, and $z = 1$ (see MS in Fig. 1 of the main text), the maximum volume for a spherical particle is

$$V = \frac{1}{8.96 \times 10^{3}\ \mathrm{kg\ m^{-3}}}\left(\frac{4.03 \times 10^{-16}\ \mathrm{C}}{96485\ \mathrm{s\ A\ mol^{-1}}}\right)\left(\frac{63.546 \times 10^{-3}\ \mathrm{kg\ mol^{-1}}}{1}\right) = 2.96 \times 10^{-26}\ \mathrm{m}^{3}, \tag{4}$$

with a corresponding maximum diameter $d_{\mathrm{max}} = 3.84$ nm. This is smaller than the majority of crystallites contained in printed structures (Supplementary Fig. 11). We thus conclude a printing mode based on growth of crystallites upon electro-reduction of cations on the substrate, as opposed to spraying of particles formed in flight.

Blocking charge transfer from the substrate

Electron transfer from the substrate is mandatory for the electrochemical growth mechanism proposed in this report. Thus, blocking the path of electrons from the substrate should interrupt growth. In contrast, interrupting electron transfer from the surface should not affect growth if ions are reduced in flight and deposited as neutralised particles. Supplementary Figure 15a outlines a corresponding experiment: a Au substrate is partially covered with an electrically insulating film (1 µm thick alumina). The Au/alumina stack effectively blocks electron transfer while at the same time assuring an unaffected electric field for EHD ejection (with the field defined as voltage applied to the source divided by the nozzle-Au distance).

When printing an array of Cu pillars across the Au-Au/alumina interface, Cu only deposits if fast electron transfer through the bare Au-layer is available (Supplementary Fig. 15b). No Cu is deposited on the oxide. Supplementary Figure 15c shows the corresponding current measured at the high-voltage output of the source: the current decreases whenever ejecting above the oxide, but does not approach zero. Thus, we conclude the EHD ejection is sustained with the nozzle positioned above the oxide. The fact that the individual positions of the array on the oxide are reflected in the current transient support that assumption: the current measured at the positions close to the edge of the oxide is always higher than at the positions at the left end of the array, presumably due to decreased charging and build up of a lower counter field at these positions. The fact that no solvent residues are visible on the oxide is probably owed to charging of the oxide and subsequent deflection of the ejected liquid.

The array shown was printed at a voltage of 110 V, but the same result was obtained in a range from 90 – 140 V. Some insignificant growth is observed for long residence times of $>$10 s and highest voltages, presumably through charge transfer from the oxide or the atmosphere. It follows that fast charge transfer from the substrate is essential for printing. Further, no neutralised particles are sprayed, else we would detect them on the oxide. In combination with the additional evidence presented in the above text, even the formation of any charged particles is unlikely, although this experiment does not exclude their formation (as they could be deflected and thus not be detected).

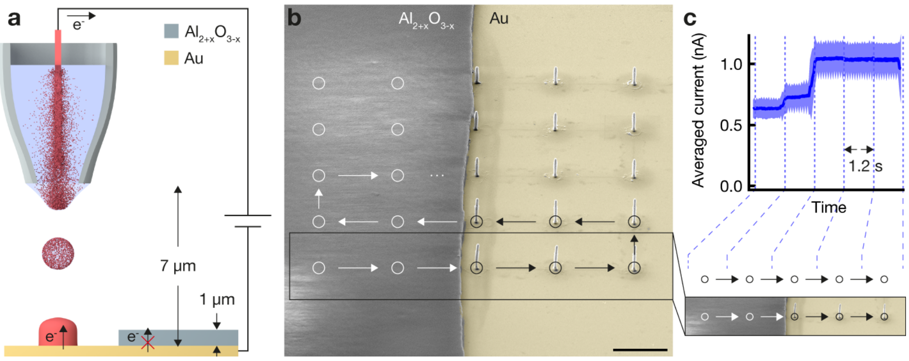

Supplementary Figure 15: **Electron transfer from the substrate is essential.** Blocking the fast electron transfer from the substrate strongly retards printing – a fact that would not be observed for any deposition modes based on particle formation in air. **a**, A 1 µm thick $Al_{2+x}O_{3-x}$ film deposited onto a Au-substrate presents an effective barrier for electrons from the substrate. Yet, a constant electric field for ejection is maintained by this substrate design. **b**, SE micrograph of an array of Cu pillars printed across the Au-Au/$Al_{2+x}O_{3-x}$ interface. The bare Au surface is false coloured in yellow, the circles mark individual positions of the array, and the arrows indicate printing direction. Although the voltage was held at 110 V during the complete array, Cu only deposits on the conductive surface. Scale bar: 5 µm. **c**, Averaged current through the source electrode during printing of an individual row of the array in (b), averaged over all rows (The solid line is the averaged data, the shading represents the standard deviation). The excerpt of the array at the bottom of (c) indicates the array positions relating to the time axis of the graph. The vertical grid indicates the residence time of 1.2 s used to print each pillar. The uninterrupted ejection current at positions on the oxide indicates ongoing ejection over the oxide.

Supplementary Methods 1: Printing Setup

The photograph in Supplementary Fig. 16 shows the printing setup as described in the methods section.

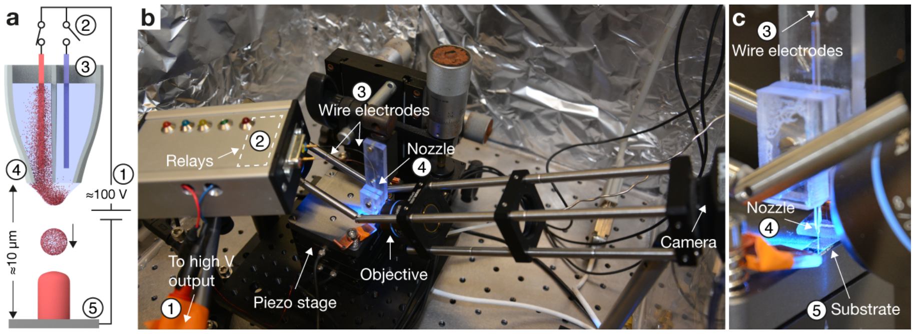

Supplementary Figure 16: **Printing setup.** **a**, Schematic of the printing process with major components of the setup labelled: (1) Voltage source, (2) mechanical relays to switch the voltage between individual electrodes, (3) metal wires used as electrodes, (4) printing nozzle, (5) grounded substrate. **b**, Main components of the printing setup, as described in the methods section. Numeric labels as in (a). **c**, Zoomed image of a two-channel nozzle positioned above the substrate, with a Cu and a Ag wire inserted into the back of the nozzle.

Supplementary Methods 2: Information to the micrographs

Supplementary Table 1: **Supplementary information to the micrographs** presented in the main text. Detection mode and tilt angle (vs. substrate normal) refer to the presented micrographs, while lateral printing speed and number of layers refer to the respective printing experiments. The printing speed and number of layers are only reported where applicable. SE: secondary electrons, BSE: backscattered electrons

Fig.	panel	detection mode	tilt angle [°]	lateral printing speed [μm s^{-1}]	num. of layers
1	b	low-vacuum SE	60	-	-
	c	optical	60	-	-
	f	SE	85	-	-
2	b	SE	0	1	1
	c	SE	87	-	-
	d	SE	87	2	39
3	a	SE	57	-	-
	b	SE	60 (top)<br>0 (bottom)	3	10 (leftmost), 3
	c	SE	0	-	-
	d	SE	82	-	-
	e	SE	85	-	-
	f	SE	34	1.5	10
	g (left)	SE	85	-	-
	g (right)	InLens SE	tilt-corrected	-	-
4	a, b	SE	60	-	-
	c	SE	82	-	-
	d – h	BSE	87	-	-

Supplementary References

[1] Galliker, P. et al. Direct printing of nanostructures by electrostatic autofocussing of ink nanodroplets. *Nat. Commun.* **3**, 890 (2012).

[2] Schneider, J. et al. Electrohydrodynamic NanoDrip Printing of High Aspect Ratio Metal Grid Transparent Electrodes. *Adv. Funct. Mater.* **26**, 833–840 (2016).

[3] Hirt, L., Reiser, A., Spolenak, R. & Zambelli, T. Additive Manufacturing of Metal Structures at the Micrometer Scale. *Adv. Mater.* **29**, 1604211 (2017).

[4] Vyatskikh, A. et al. Additive manufacturing of 3D nano-architected metals. *Nat. Commun.* **9**, 593 (2018).

[5] Lee, J.-S. et al. Design and evaluation of a silicon based multi-nozzle for addressable jetting using a controlled flow rate in electrohydrodynamic jet printing. *Appl. Phys. Lett.* **93**, 243114 (2008).

[6] Pan, Y., Chen, X., Zeng, L., Huang, Y. & Yin, Z. Fabrication and evaluation of a protruding Si-based printhead for electrohydrodynamic jet printing. *J. Micromechanics Microengineering* **27**, 125004 (2017).

[7] Jaworek, A. & Krupa, A. Classification of the modes of EHD spraying. *J. Aerosol Sci.* **30**, 873–893 (1999).

[8] Lee, A., Jin, H., Dang, H. W., Choi, K. H. & Ahn, K. H. Optimization of experimental parameters to determine the jetting regimes in electrohydrodynamic printing. *Langmuir* **29**, 13630–13639 (2013).

[9] Chen, J., Lu, L. & Lu, K. Hardness and strain rate sensitivity of nanocrystalline Cu. *Scr. Mater.* **54**, 1913–1918 (2006).

[10] Chang, S.-Y. & Chang, T.-K. Grain size effect on nanomechanical properties and deformation behavior of copper under nanoindentation test. *J. Appl. Phys.* **101**, 033507 (2007).

[11] Bass, J. 1.2.1 Pure metal resistivities at T = 273.2 K. In K.-H. Hellwege & J. L. Olsen, editors, *Electr. Resist. Kondo Spin Fluct. Syst. Spin Glas. Thermopower*, pages 5–13, (Springer-Verlag, Berlin/Heidelberg1983).

[12] Rossnagel, S. M. & Kuan, T. S. Alteration of Cu conductivity in the size effect regime. *J. Vac. Sci. Technol. B Microelectron. Nanom. Struct.* **22**, 240 (2004).

[13] Tuck, D. G. Direct electrochemical synthesis of inorganic and organometallic compounds. *Pure Appl. Chem.* **51**, 2005–2018 (1979).

[14] Vecchio-Sadus, A. M. The electrochemical synthesis of inorganic and organometallic complexes in non-aqueous media. *J. Appl. Electrochem.* **23**, 401–416 (1993).

[15] Berkel, G. J., Asano, K. G. & Schnier, P. D. Electrochemical processes in a wire-in-a-capillary bulk-loaded, nano-electrospray emitter. *J. Am. Soc. Mass Spectrom.* **12**, 853–862 (2001).

[16] Prudent, M. & Girault, H. H. Functional electrospray emitters. *Analyst* **134**, 2189 (2009).

[17] Li, A., Luo, Q., Park, S.-J. & Cooks, R. G. Synthesis and Catalytic Reactions of Nanoparticles formed by Electrospray Ionization of Coinage Metals. *Angew. Chemie Int. Ed.* **53**, 3147–3150 (2014).

[18] Li, A. et al. Using ambient ion beams to write nanostructured patterns for surface enhanced Raman spectroscopy. *Angew. Chem. Int. Ed. Engl.* **53**, 12528–31 (2014).

[19] Jaworek, A. & Sobczyk, A. Electrospraying route to nanotechnology: An overview. *J. Electrostat.* **66**, 197–219 (2008).

[20] Duft, D., Achtzehn, T., Müller, R., Huber, B. A. & Leisner, T. Rayleigh jets from levitated microdroplets. *Nature* **421**, 128–128 (2003).

[21] Fernández de la Mora, J. The Fluid Dynamics of Taylor Cones. *Annu. Rev. Fluid Mech.* **39**, 217–243 (2007).

[22] Onses, M. S., Sutanto, E., Ferreira, P. M., Alleyne, A. G. & Rogers, J. A. Mechanisms, Capabilities, and Applications of High-Resolution Electrohydrodynamic Jet Printing. *Small* **11**, 4237–4266 (2015).

[23] Smallwood, I. *Handbook of Organic Solvent Properties*, (Elsevier1996).
