# Supplementary material for: Multi-metal electrohydrodynamic redox 3D printing at the submicron scale
Source: Nat Commun. 2019 Apr 23;10:1853. doi: 10.1038/s41467-019-09827-1 (PMC6479051; doi:10.1038/s41467-019-09827-1)
Supplement: Supplementary file 3 — Description of Additional Supplementary Files [file 41467_2019_9827_MOESM3_ESM.pdf]

Description of Additional Supplementary Files

File Name: Supplementary Movie 1
Description: On-the-fly switching between Cu and Ag. Optical video of the printing process of a Cu wall with the letters ’Ag’ imprinted in silver (as shown in Fig. 2d of the main text). On-the-fly alteration of two metals ejected from the same nozzle enables printing of chemically heterogeneous structures in a continuous layer-by-layer mode. The fast switching between the two materials facilitates a spatio-chemical feature size of <400 nm obtained with minimal pulse width of 100 ms and a printing speed of 2 $\mu m\ s^{-1}$. The two LEDs in the upper left corner indicate which material is printed: they are connected to the mechanical relays switching the high voltage between the two electrodes used as ion sources, and thus signal, which electrode is currently biased. Note: The shown SEM micrographs were not recorded from the wall seen in the video, but from a wall printed onto the same sample with the same nozzle and the same printing and switching speed. Video playback speed: 1x; tilt: 60° vs. substrate normal.

File Name: Supplementary Movie 2
Description: Printing a diffraction grating. Optical video of the printing of 300 x 300 Cu pillars that build a diffraction grating 450 x 450 μm^2 in size. Optical dark field images at the end of the video show the first order of diffraction of the grating. Video playback speed: 1x; tilt: 60° vs. substrate normal.

File Name: Supplementary Movie 3
Description: Locally tuned strength through gradient porosity. Additive control of the chemical architecture enables tuning of local properties through the local chemistry. The video demonstrates the fabrication of pillars with a porosity gradient modulating the local strength: first, segmented Ag / Cu-Ag pillars are printed. The Cu-Ag alloy contains 45 – 50 at.% Cu. Subsequently, the Cu is chemically dealloyed from the Cu-Ag segments, resulting in Ag pillars that are half dense, half nanoporous. Since the porous segments are softer than the dense segments, lateral deformation of the pillars results in a strong confinement of the plasticity within the porous portions of the pillars. The bending of the pillars is demonstrated in an in situ SEM video. Video playback speeds: printing: 1x, bending: 20x and 11.45x; tilt: printing: 60°, bending: 70° vs. substrate normal.
